# An enhanced computational platform for investigating the roles of regulatory RNA and for identifying functional RNA motifs

**DOI:** 10.1186/1471-2105-14-S2-S4

**Published:** 2013-01-21

**Authors:** Tzu-Hao Chang, Hsi-Yuan Huang, Justin Bo-Kai Hsu, Shun-Long Weng, Jorng-Tzong Horng, Hsien-Da Huang

**Affiliations:** 1 Graduate Institute of Biomedical Informatics, Taipei Medical University, Taipei, Taiwan; 2 Institute of Bioinformatics and Systems Biology, National Chiao Tung University, Hsin-Chu 300, Taiwan; 3 Department of Biological Science and Technology, National Chiao Tung University, Hsin-Chu 300, Taiwan; 4 Department of Obstetrics and Gynecology, Hsinchu Mackay Memorial Hospital, Hsinchu, Taiwan; 5 Mackay Medicine, Nursing and Management College, Taipei, Taiwan; 6 Department of Medicine, Mackay Medical College, New Taipei City, Taiwan; 7 Department of Computer Science and Information Engineering, National Central University, Chung-Li 320, Taiwan; 8 Institute of Systems Biology and Bioinformatics, National Central University, Chung-Li 320, Taiwan; 9 Department of Biomedical informatics, Asia University, Wufeng 413, Taiwan

## Abstract

**Background:**

Functional RNA molecules participate in numerous biological processes, ranging from gene regulation to protein synthesis. Analysis of functional RNA motifs and elements in RNA sequences can obtain useful information for deciphering RNA regulatory mechanisms. Our previous work, RegRNA, is widely used in the identification of regulatory motifs, and this work extends it by incorporating more comprehensive and updated data sources and analytical approaches into a new platform.

**Methods and results:**

An integrated web-based system, RegRNA 2.0, has been developed for comprehensively identifying the functional RNA motifs and sites in an input RNA sequence. Numerous data sources and analytical approaches are integrated, and several types of functional RNA motifs and sites can be identified by RegRNA 2.0: (i) splicing donor/acceptor sites; (ii) splicing regulatory motifs; (iii) polyadenylation sites; (iv) ribosome binding sites; (v) rho-independent terminator; (vi) motifs in mRNA 5'-untranslated region (5'UTR) and 3'UTR; (vii) AU-rich elements; (viii) C-to-U editing sites; (ix) riboswitches; (x) RNA cis-regulatory elements; (xi) transcriptional regulatory motifs; (xii) user-defined motifs; (xiii) similar functional RNA sequences; (xiv) microRNA target sites; (xv) non-coding RNA hybridization sites; (xvi) long stems; (xvii) open reading frames; (xviii) related information of an RNA sequence. User can submit an RNA sequence and obtain the predictive results through RegRNA 2.0 web page.

**Conclusions:**

RegRNA 2.0 is an easy to use web server for identifying regulatory RNA motifs and functional sites. Through its integrated user-friendly interface, user is capable of using various analytical approaches and observing results with graphical visualization conveniently. RegRNA 2.0 is now available at http://regrna2.mbc.nctu.edu.tw.

## Background

Numerous functional RNA motifs have been identified as playing significant roles in many essential biological processes, including transcriptional and post-transcriptional regulation of gene expression, control of mRNA stability, alternative splicing, and transcription termination. The biological activities of functional RNA motifs usually rely on a combination of their primary sequences and specific secondary structures, which act as target sites of RNA-binding factors or directly interact with translation machinery [1]. For instance, riboswitches are metabolite-binding domain within a specific mRNA, and can regulate both transcription and translation by binding their corresponding targets [2,3].

Several databases were established for collecting functional RNA molecules [1,4-14]. UTRdb [1] is a database of 5' and 3' untranslated sequences of eukaryotic mRNAs. It provides specialized information including the presence of nucleotide sequence patterns already demonstrated by experimental analysis to have some functional roles, and these patterns have been collected into the UTRsite database. Rfam [4,5] is a database comprehensively collecting families of non-coding RNA (ncRNA) genes as well as *cis*-regulatory RNA elements. Each family is represented by a multiple sequence alignment of known and predicted representative members, and annotated with a consensus base-paired secondary structure. It facilitates the identification and classification of new members of known RNA families, and provides the glimpses of conservation of multiple ncRNA families across a wide taxonomic range. fRNAdb [6,7] is a database hosting a large collection of ncRNA sequence data from public non-coding databases, and provides related annotations, such as sequence ontology classification and source organisms. AEdb is a database for alternative exons and their properties from numerous species, and it forms the manually curated component of alternative splicing database (ASD) [8]. The data in AEdb is gathered from literature where these exons have been experimentally verified. The adenylate uridylate-rich elements (AREs or AU-rich element) mediate the rapid turnover of mRNA encoding proteins that regulate cellular growth and body response to exogenous agent such as microbes and environmental stimuli. ARED [9,10] is a human AU-rich element-containing mRNA database. A 13-bp ARE pattern was computationally derived using MEME, and five clusters were generated from ARE sequences. NONCODE [11,12] is an integrated knowledge database designed for analysis of ncRNAs. All ncRNAs in NONCODE were confirmed by consulting the references manually and more than 80% data are from experiments. microRNAs (miRNAs) are small RNA molecules, which are ~22 nt sequences, and participate in gene post-transcriptional regulation and degradation of mRNA by hybridizing to miRNA target sites. miRBase [13] is the central online repository for miRNA nomenclature, sequence data annotation and target prediction. It provides a range of data to facilitate studies of miRNA genomics. TRANSFAC [14] is a knowledge-base containing published data on eukaryotic transcription factors, their experimentally-proven binding site, and regulated genes.

Various approaches were developed for identifying functional RNA motifs or elements [15-26]. GeneSplicer [15] was developed for detecting splice sites in eukaryotic mRNA by combining several techniques, such as maximal dependence decomposition (MDD) and Markov model, that have already proven successful in characterizing the patterns around the donor and acceptor sites. polya_svm [16] was developed for predicting mRNA polyadenylation site using a Support Vector Machine (SVM) featuring 15 over-represented *cis*-regulatory elements in various regions surrounding. RBSfinder [17] is a probabilistic method to improve the accuracy of gene identification systems at finding precise translation start sites. TransTermHP [18] can rapidly and accurately detecting rho-independent transcription terminators. CURE [19] was developed for predicting C-to-U RNA editing site in plant mitochondria by incorporating both evolutionary and biochemical information. miRanda [20] was developed for finding genomic targets for miRNAs. RiboSW [21] is a systematic method for identifying 12 kinds of riboswitches based on RNA conserved functional sequences and conformations. PatSearch [22] was developed for searching specific combinations of oligonucleotide consensus sequences, secondary structure motifs and position-weight matrices (PWMs). ERPIN [23] is a practical approach for the automatic derivation of an RNA signature from a sequence alignment and secondary structure, and finding the occurrence in sequence databases. Several profiles have been constructed to search any input sequence for the presence of some RNA genes and elements on ERPIN web server. INFERNAL [24] is an implementation of a general stochastic context-free grammars (SCFG) based approach for RNA database searches and multiple alignment. It is used to annotate RNAs in genomes in conjunction with the Rfam families by covariance models, a special case of SCFGs designed for modeling RNA consensus sequence and structure. MATCH [25] is an approach for searching transcription factor binding sites with specific position-weight matrices (PWM). RNAMotif [26] is an RNA secondary structure definition and search algorithm, and commonly used for searching user-defined RNA motifs.

Analysis of functional RNA motifs and sites in RNA sequences can obtain useful information for deciphering RNA regulatory mechanisms. Our previous work, RegRNA [27], is widely used to identify regulatory motifs and miRNA target sites, and has been cited 50 times. However, various types of functional RNA motifs and identification approaches were continuously accumulated and developed in recent years. In order to comprehensively identify functional RNA motifs, a more complete and updated analysis platform is crucial.

This work presents an integrated web server, RegRNA 2.0, for identifying functional RNA motifs and sites in an input RNA sequence. Numerous data sources, such as Rfam [4], fRNAdb [6] and UTRsite [1], and identification approaches, such as GeneSplicer [15], RiboSW [21] and RBSfinder [17], were integrated in RegRNA 2.0, and other additional information, such as GC-content ratio and RNA accessibility, are also presented on the web page. User can submit an RNA sequence through our user-friendly interface, and obtain the predictive results with graphical visualization.

## Methods

### Data collection

The functional RNA motifs and sites supported in RegRNA 2.0 are categorized into several types: (i) splicing donor/acceptor sites; (ii) splicing regulatory motifs; (iii) polyadenylation sites; (iv) ribosome binding sites; (v) rho-independent terminator; (vi) motifs in mRNA 5'-untranslated region (5'UTR) and 3'-UTR; (vii) AU-rich elements; (viii) C-to-U editing sites; (ix) riboswitches; (x) RNA cis-regulatory elements; (xi) transcriptional regulatory motifs; (xii) user-defined motifs; (xiii) similar functional RNA sequences; (xiv) microRNA target sites; (xv) non-coding RNA hybridization sites; (xvi) long stems; (xvii) open reading frames; (xviii) related information of an RNA sequence.

**Figure 1:**
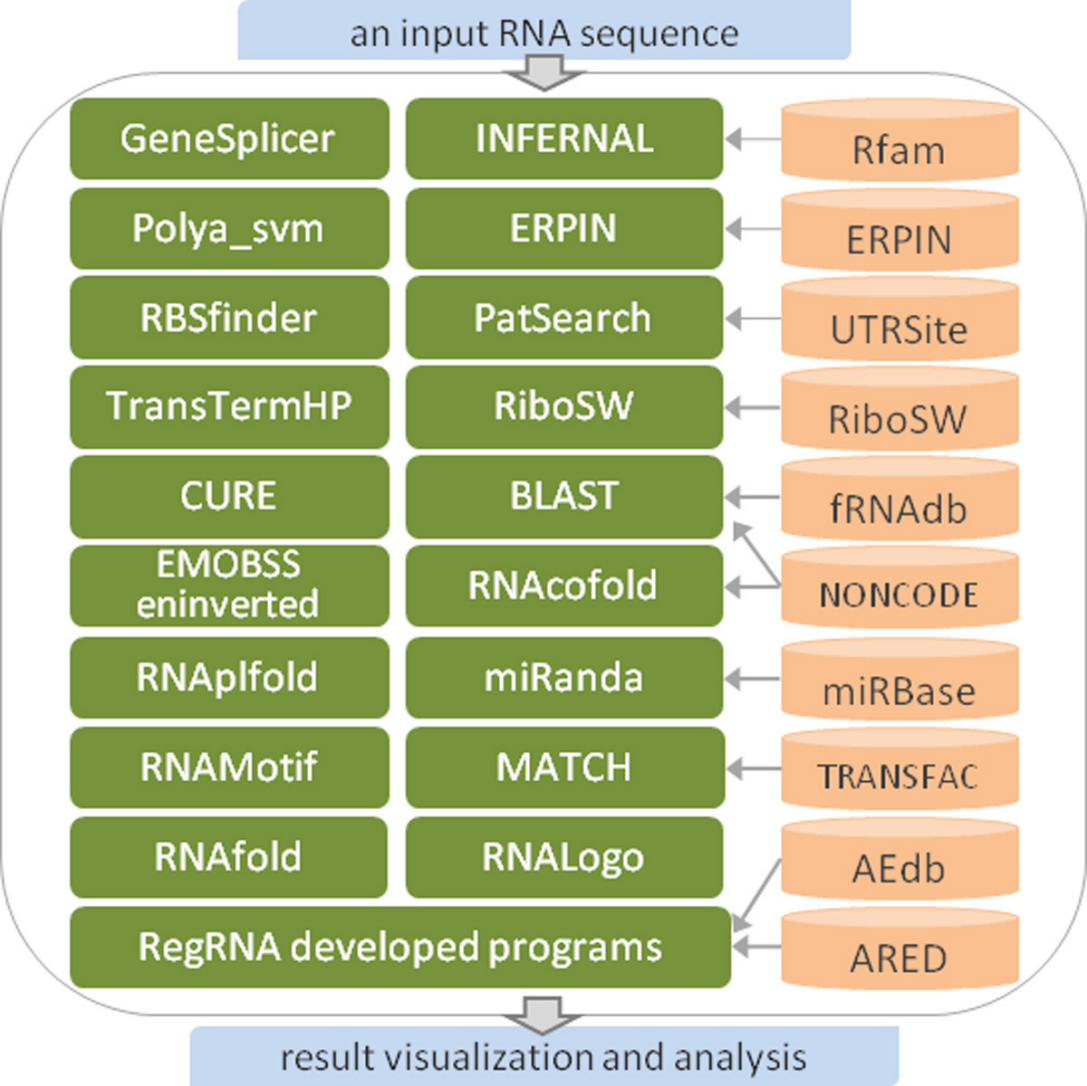
**The process flow of RegRNA 2.0**.

The process flow of RegRNA 2.0 is depicted in Figure 1. Numerous functional RNA motifs and sites were collected from a variety of databases and websites including Rfam [4], ERPIN [23], RiboSW [21], UTRsite [1], AEdb [8], ARED [10], fRNAdb [7], NONCODE [11], miRBase [28] and TRANSFAC [14]. As shown in Table 1, different prediction models, sequences and patterns are incorporated into RegRNA 2.0. There are 209 covariance models (CMs) of Rfam *cis*-regulatory families, 11 profiles of ERPIN RNA elements, 12 descriptors of RiboSW riboswitches, 48 models of UTRsite motifs and 2,171 transcription factor binding matrices of TRNASFAC, 294 sequence patterns of AEdb splicing regulatory motifs, 5 sequence patterns of ARED AU-rich elements, 475,318 fRNAdb sequences whose length are less than 500 nt, 21,643 miRNA sequences of miRBase and 170,581 ncRNA sequences of NONCODE collected for identifying different functional RNA motifs and sites.

**Table 1:** Statistics of types of functional RNA motifs supported in RegRNA 2.0

Types of functional RNA motifs	Incorporated data	Incorporated approaches	Number of entries
Splicing sites		GeneSplicer [15]	
Splicing regulatory motifs	AEdb [8]	RegRNA 2.0	294 splicing motifs
Polyadenylation Sites		polya_svm [16]	
Ribosome binding site		RBSfinder [17]	
Rho-independent terminator		TransTermHP [18]	
UTR Motifs	UTRsite [1]	PatSearch [22]	48 UTRsite motifs
AU-rich element	ARED [10]	RegRNA 2.0	5 ARE patterns
RNA editing sites		CURE [19]	
Riboswitches	RiboSW models	RiboSW [21]	12 riboswitches
RNA elements	ERPIN profiles	ERPIN [23]	11 RNA elements
RNA *cis*-regulatory elements	Rfam CMs [4]	INFERNAL [24]	209 Rfam *cis*-reg families
Long stem		einverted [30]	
Known functional RNAs	fRNAdb [7]	BLAST [29]	475,318 fRNAdb sequences
miRNA target	miRBase [28]	miRanda [20]	21,643 miRNA sequences
ncRNA hybridization sites	NONCODE [11]	BLAST [29], RNAcofold [34]	170,581 ncRNA sequences
TRANSFAC motifs	TRANSFAC [14]	Match [25]	2,171 transcription factor binding matrices
User-defined motif		RNAMotif [26]	
Open Reading Frame		RegRNA 2.0	
GC-content ratio		RegRNA 2.0	
RNA accessibility		RNAplfold [34]	

### Development of identification procedures

Numerous analytical approaches and data sources were integrated in RegRNA 2.0 (Table 1). GeneSplicer [15], polya_svm [16], RBSfinder [17], TransTermHP [18], CURE [19], RiboSW [21], and ERPIN [23], are incorporated for identifying splicing sites, polyadenylation sites, ribosome binding sites, Rho-independent terminator, C-to-U editing sites, riboswitches, and RNA elements, respectively. MATCH [25] is used with matrices collected in TRANSFAC [14] to provide the possibility to search for a variety of different transcription factor finding sites. PatSearch [22] and UTRsite models are integrated for indentifying UTR motifs. INFERNAL [24] and Rfam CMs are integrated for identifying *cis*-regulatory families. miRanda [20] and miRNA sequences of miRBase are integrated for identifying miRNA target sites. BLAST [29] and sequences of fRNAdb is integrated for finding similar functional RNA sequences. The einverted of EMBOSS package [30] is utilized for identifying long stems, which might be involved in mechanisms of gene regulatory processes [31-33]. For identifying putative RNA-RNA interaction sites, BLAST is used to find the complementary subsequence of input sequence against NONCODE database, and RNAcofold of Vienna RNA Package [34] is used to compute the free energy of hybridization sites. RNAMotif [26] is integrated for searching user-defined RNA motifs. In addition, RegRNA 2.0 is capable of predicting ORFs of the input RNA sequence. The default options are for resulting protein of at least 80 amino acids beginning with a start codon (AUG, GUG or UUG) and ending with a stop codon (UAA, UAG or UGA). The fully overlapped ORFs are not shown. Other related information, such as GC-content ratio and RNA accessibility, are also provided for the input RNA sequence. RNAplfold and RNAfold of Vienna RNA package [34] are used for predicting RNA accessibility and RNA secondary structure, respectively.

### User interface

**Figure 2:**
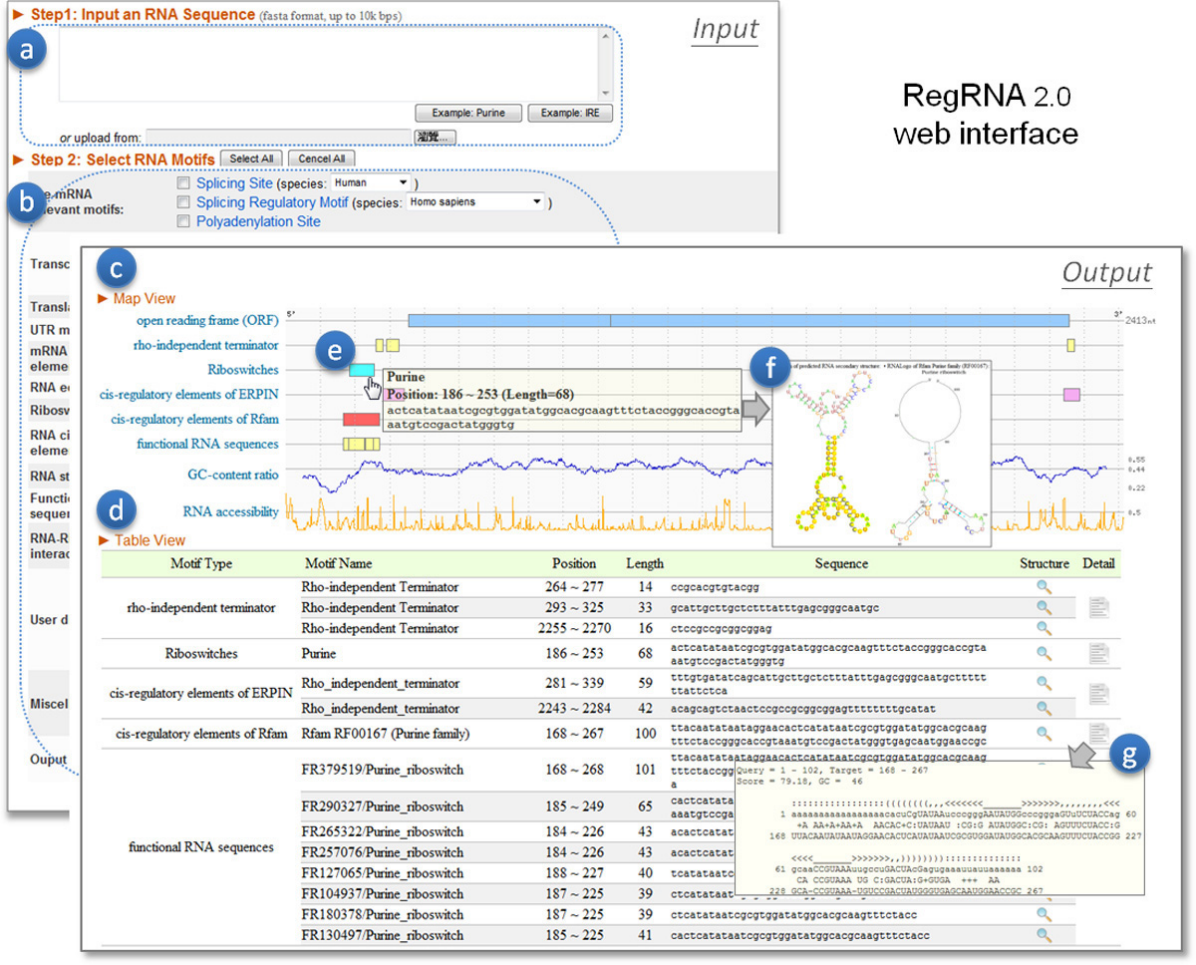
**The RegRNA 2.0 web interface (a) input area (b) parameter setting (c) map view (d) table view (e) motif information (f) structure information (g) details**.

An integrated web-based system with user-friendly interface (Figure 2) was developed to facilitate user conveniently and comprehensively identifying functional RNA motifs and sites in an RNA sequence. User can submit a sequence by inputting a single sequence in FASTA format, or uploading a sequence file (Figure 2a), and the predictive results are presented via a graphical interface. User can decide which types of functional RNA motifs to be investigated by clicking the checkbox (Figure 2b). All parameters were set with default values, and user can alter the thresholds to fit their requirement. For instance, in predicting miRNA target sites, users can select the specie and adjust the minimum free energy (MFE) threshold and score threshold to filter miRNA targets of interest.

RegRNA 2.0 provides an intuitive graphical visualization (map view, Figure 2c) and summarized information table (table view, Figure 2d) for predictive results. The graphic location maps are created for intuitively displaying the positions of predictive motifs. The top-most graph shows the predictive ORFs, and the following graphs shows the predictive functional RNA motifs or sites. User can see the brief introduction of a predictive motif, such as the name, the start/end positions and the binding factors, by moving the cursor on it, and a pop-up description will be shown on the screen directly (Figure 2e). Further analysis and additional information of a predictive motif, such as the predictive secondary structure and the corresponding RNALogo [35] graph, can be observed by clicking on the motifs of interest (Figure 2f). The details of predictive results can be obtained in summarized information table (Figure 2g).

## Results

### A case study of identification of purine riboswitch

The purine riboswitch is used as a case study to demonstrate the capability of RegRNA 2.0. Purine riboswitches, which are found in the 5'UTR of mRNAs act as cis-acting genetic regulatory elements composed of a metabolite-responsive aptamer domain in a specific secondary structure. It can regulate both transcription and translation by binding their corresponding targets. Additional file 1 illustrates a cartoon representation of the mechanism of genetic regulation by the guanine riboswitch [36]. In the presence high concentrations of guanine or hypoxanthine, ligand binding stabilizes the three-way junction structure, allowing the mRNA to form the terminator element (cyan). Without ligand binding, the 3'side of the P1 stem (green) and the 5'side of the terminator are used to form an antiterminator element, allowing transcription to continue.

An RNA sequence with the accession number of EMBL, X83878, was used as an input for RegRNA 2.0. There exist a purine riboswitch and an operon of two genes, *B. subtilis xpt *and *pubX*, in X83878 according to the annotations of Rfam and EMBL database. The total length of X83878 is 2413 bps, and the location of purine riboswitch is from position 168 to 276. The location of CDS regions of *xpt *and *pubX *are from position 357 to 941 and from position 938 to 2254, respectively.

**Figure 3:**
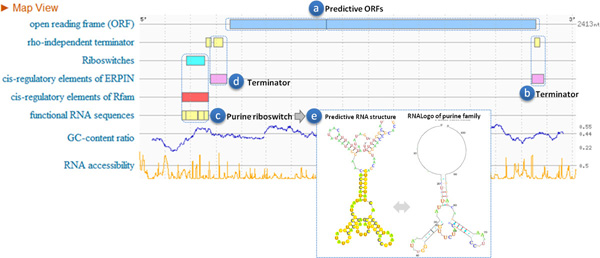
**The RegRNA 2.0 predictive results of an input sequence, X83878, which is annotated with an operon of two genes and a purine riboswitch in EMBL and Rfam, respectively**. (a) predictive ORFs (b) terminator (c) purine riboswitch (d) terminator (e) predictive structure and RNALogo of purine family.

Figure 3 shows the RegRNA 2.0 predictive results of the case study. The locations of two CDS regions were correctly predicted (Figure 3a), and the terminator of this operon was recognized by two RegRNA 2.0 identification procedures, TransTermHP and ERPIN (Figure 3b). The location of purine riboswitch was identified by three RegRNA 2.0 identification procedures, RiboSW, INFERNAL and BLAST fRNAdb (Figure 3c). A crucial RNA secondary structure, terminator, for purine riboswitch regulating gene activity was also predicted (Figure 3d), and the location of this terminator is close following the predictive purine riboswitch that corresponds to the mechanism of genetic regulation of the purine riboswitch [36]. In addition, the MFE secondary structure of predictive purine riboswitch regions shows the similar conformation to the RNALogo graph of Rfam purine family (Figure 3e). The results of case study show that RegRNA 2.0 is capable of identifying and displaying useful information in a given RNA sequence, and helpful for observing and deciphering RNA regulatory mechanisms.

## Discussions and conclusions

RegRNA 2.0 facilitates user to identify functional RNA motifs and sites in an RNA sequence. As compared with our previously work, RegRNA [27], RegRNA 2.0 incorporates more data sources and analytical approaches (Table 2). RegRNA 2.0 enables user to identify more types of functional RNA motifs and sites including polyadenylation sites, ribosome binding sites, rho-independent terminator, AU-rich elements, RNA editing sites, RNA cis-regulatory elements, similar functional RNA sequences, non-coding RNA hybridization sites, long stems, open reading frames and related information of an RNA sequence. Additionally, RegRNA 2.0 provides further analysis, such as RNA secondary structure, RNA accessibility and RNALogo graph, for the predictive results, and display results with intuitive graphical visualization.

**Table 2:** A comparison between RegRNA 2.0 and RegRNA

Features	**RegRNA **[27]	RegRNA 2.0
Polyadenylation sites	-	**Yes **(polya_svm)
Ribosome binding sites	-	**Yes **(RBSfinder)
Rho-independent terminator	-	**Yes **(TransTermHP)
RNA editing sites	-	**Yes **(CURE)
AU-rich elements	-	**Yes **(ARED)
RNA *cis*-elements	-	**Yes **(Rfam & ERPIN)
similar functional RNAs	-	**Yes **(BLAST+fRNAdb)
ncRNA hybridization region	-	**Yes **(BLAST+RNAcofold+NONCODE)
Open reading frame	-	**Yes **(RegRNA 2.0)
Motif region structure	-	**Yes **(RNAfold)
RNALogo displaying	-	**Yes **(RNALogo)
GC-content Ratio	-	**Yes **(RegRNA 2.0)
RNA accessibility	-	**Yes **(RNAplfold)
DNA motifs	Yes (TRANSFAC 7.4)	**Yes & Updated **(TRANSFAC 2012.1)
Splicing regulatory motifs	Yes (AEDB 278 motifs)	**Yes & Updated **(AEDB 294 motifs)
UTR motifs	Yes (UTRSite 40 motifs)	**Yes & Updated **(UTRSite 48 motifs)
Riboswitches	Yes (RNAMotif)	**Yes & Updated **(RiboSW & Rfam)
miRNA target sites	Yes (744 miRNAs)	**Yes & Updated **(miRBase 21,643 miRNAs)
Splicing sites	Yes	**Yes **(GeneSplicer)
Long stems	Yes	**Yes **(EMBOSS einverted)
User-defined Motifs	Yes	**Yes **(RNAMotif)

RegRNA 2.0 is an easy to use web server for comprehensively identifying regulatory RNA motifs and functional sites. It extends the widely used analysis platform, RegRNA [27], by taking more types of motifs and analytical approaches into consideration. RegRNA 2.0 is convenient to use programs without having to download the code and get the programs to run. Through its integrated user-friendly interface, user is capable of using various analytical approaches and observing results with graphical visualization conveniently. The platform will be enhanced by supporting input of multiple RNA sequences and providing conservation analysis in the future.

## Availability and requirements

The RegRNA 2.0 system is freely available at http://regrna2.mbc.nctu.edu.tw.

## Competing interests

The authors declare that they have no competing interests.

## Authors' contributions

JTH and HDH conceived and supervised the study. THC, HYH, JBKH and SLW were responsible for the design, computational analyses, implementation of the system, and drafting the manuscript. All authors read and approved the final manuscript.

## Declarations

The authors approved the submission of this paper to *BMC Bioinformatics* for publication. The payment of publishing charges to BioMed Central for this article was supported by National Science Council of the Republic of China, No. NSC 101-2311-B-009-003-MY3 and NSC 100-2627-B-009-002. This publishing charge was supported in part by the UST-UCSD International Center of Excellence in Advanced Bio-engineering sponsored by the Taiwan National Science Council I-RiCE Program under Grant Number: NSC 101-2911-I-009-101, and Veterans General Hospitals and University System of Taiwan (VGHUST) Joint Research Program under Grant Number: VGHUST101-G5-1-1. This publishing charge is also partially supported by MOE ATU.

This article has been published as part of *BMC Bioinformatics *Volume 14 Supplement 2, 2013: Selected articles from the Eleventh Asia Pacific Bioinformatics Conference (APBC 2013): Bioinformatics. The full contents of the supplement are available online at http://www.biomedcentral.com/bmcbioinformatics/supplements/14/S2.

## Supplementary Material

Additional file 1 **A cartoon representation of the mechanism of genetic regulation by the guanine riboswitch **[**36**]. Click here for file

## References

[B1] Mignone F Grillo G Licciulli F Iacono M Liuni S Kersey PJ Duarte J Saccone C Pesole G UTRdb and UTRsite: a collection of sequences and regulatory motifs of the untranslated regions of eukaryotic mRNAs Nucleic acids research 2005 33 Database D141 146 15608165 10.1093/nar/gki021 PMC539975

[B2] Roth A Breaker RR The structural and functional diversity of metabolite-binding riboswitches Annu Rev Biochem 2009 78 305 334 10.1146/annurev.biochem.78.070507.135656 19298181 PMC5325118

[B3] Montange RK Batey RT Riboswitches: emerging themes in RNA structure and function Annu Rev Biophys 2008 37 117 133 10.1146/annurev.biophys.37.032807.130000 18573075

[B4] Gardner PP Daub J Tate JG Nawrocki EP Kolbe DL Lindgreen S Wilkinson AC Finn RD Griffiths-Jones S Eddy SR Rfam: updates to the RNA families database Nucleic acids research 2009 37 Database D136 140 10.1093/nar/gkn766 18953034 PMC2686503

[B5] Griffiths-Jones S Moxon S Marshall M Khanna A Eddy SR Bateman A Rfam: annotating non-coding RNAs in complete genomes Nucleic acids research 2005 33 Database D121 124 15608160 10.1093/nar/gki081 PMC540035

[B6] Mituyama T Yamada K Hattori E Okida H Ono Y Terai G Yoshizawa A Komori T Asai K The Functional RNA Database 3.0: databases to support mining and annotation of functional RNAs Nucleic acids research 2009 37 Database D89 92 10.1093/nar/gkn805 18948287 PMC2686472

[B7] Kin T Yamada K Terai G Okida H Yoshinari Y Ono Y Kojima A Kimura Y Komori T Asai K fRNAdb: a platform for mining/annotating functional RNA candidates from non-coding RNA sequences Nucleic acids research 2007 35 Database D145 148 10.1093/nar/gkl837 17099231 PMC1669753

[B8] Stamm S Riethoven JJ Le Texier V Gopalakrishnan C Kumanduri V Tang Y Barbosa-Morais NL Thanaraj TA ASD: a bioinformatics resource on alternative splicing Nucleic acids research 2006 34 Database D46 55 16381912 10.1093/nar/gkj031 PMC1347394

[B9] Bakheet T Frevel M Williams BR Greer W Khabar KS ARED: human AU-rich element-containing mRNA database reveals an unexpectedly diverse functional repertoire of encoded proteins Nucleic acids research 2001 29 1 246 254 10.1093/nar/29.1.246 11125104 PMC29778

[B10] Bakheet T Williams BR Khabar KS ARED 2.0: an update of AU-rich element mRNA database Nucleic acids research 2003 31 1 421 423 10.1093/nar/gkg023 12520039 PMC165470

[B11] He S Liu C Skogerbo G Zhao H Wang J Liu T Bai B Zhao Y Chen R NONCODE v2.0: decoding the non-coding Nucleic acids research 2008 36 Database D170 172 18000000 10.1093/nar/gkm1011 PMC2238973

[B12] Liu C Bai B Skogerbo G Cai L Deng W Zhang Y Bu D Zhao Y Chen R NONCODE: an integrated knowledge database of non-coding RNAs Nucleic acids research 2005 33 Database D112 115 15608158 10.1093/nar/gki041 PMC539995

[B13] Griffiths-Jones S miRBase: the microRNA sequence database Methods Mol Biol 2006 342 129 138 16957372 10.1385/1-59745-123-1:129

[B14] Matys V Fricke E Geffers R Gossling E Haubrock M Hehl R Hornischer K Karas D Kel AE Kel-Margoulis OV TRANSFAC: transcriptional regulation, from patterns to profiles Nucleic acids research 2003 31 1 374 378 10.1093/nar/gkg108 12520026 PMC165555

[B15] Pertea M Lin X Salzberg SL GeneSplicer: a new computational method for splice site prediction Nucleic acids research 2001 29 5 1185 1190 10.1093/nar/29.5.1185 11222768 PMC29713

[B16] Cheng Y Miura RM Tian B Prediction of mRNA polyadenylation sites by support vector machine Bioinformatics 2006 22 19 2320 2325 10.1093/bioinformatics/btl394 16870936

[B17] Suzek BE Ermolaeva MD Schreiber M Salzberg SL A probabilistic method for identifying start codons in bacterial genomes Bioinformatics 2001 17 12 1123 1130 10.1093/bioinformatics/17.12.1123 11751220

[B18] Kingsford CL Ayanbule K Salzberg SL Rapid, accurate, computational discovery of Rho-independent transcription terminators illuminates their relationship to DNA uptake Genome Biol 2007 8 2 R22 10.1186/gb-2007-8-2-r22 17313685 PMC1852404

[B19] Du P Li Y Prediction of C-to-U RNA editing sites in plant mitochondria using both biochemical and evolutionary information J Theor Biol 2008 253 3 579 586 10.1016/j.jtbi.2008.04.006 18511083

[B20] John B Enright AJ Aravin A Tuschl T Sander C Marks DS Human MicroRNA targets PLoS Biol 2004 2 11 e363 10.1371/journal.pbio.0020363 15502875 PMC521178

[B21] Chang TH Huang HD Wu LC Yeh CT Liu BJ Horng JT Computational identification of riboswitches based on RNA conserved functional sequences and conformations Rna 2009 15 7 1426 1430 10.1261/rna.1623809 19460868 PMC2704089

[B22] Grillo G Licciulli F Liuni S Sbisa E Pesole G PatSearch: A program for the detection of patterns and structural motifs in nucleotide sequences Nucleic acids research 2003 31 13 3608 3612 10.1093/nar/gkg548 12824377 PMC168955

[B23] Gautheret D Lambert A Direct RNA motif definition and identification from multiple sequence alignments using secondary structure profiles J Mol Biol 2001 313 5 1003 1011 10.1006/jmbi.2001.5102 11700055

[B24] Nawrocki EP Kolbe DL Eddy SR Infernal 1.0: inference of RNA alignments Bioinformatics 2009 25 10 1335 1337 10.1093/bioinformatics/btp157 19307242 PMC2732312

[B25] Kel AE Gossling E Reuter I Cheremushkin E Kel-Margoulis OV Wingender E MATCH: A tool for searching transcription factor binding sites in DNA sequences Nucleic acids research 2003 31 13 3576 3579 10.1093/nar/gkg585 12824369 PMC169193

[B26] Macke TJ Ecker DJ Gutell RR Gautheret D Case DA Sampath R RNAMotif, an RNA secondary structure definition and search algorithm Nucleic acids research 2001 29 22 4724 4735 10.1093/nar/29.22.4724 11713323 PMC92549

[B27] Huang HY Chien CH Jen KH Huang HD RegRNA: an integrated web server for identifying regulatory RNA motifs and elements Nucleic acids research 2006 34 Web Server W429 434 10.1093/nar/gkl333 16845041 PMC1538840

[B28] Griffiths-Jones S Saini HK van Dongen S Enright AJ miRBase: tools for microRNA genomics Nucleic acids research 2008 36 Database D154 158 17991681 10.1093/nar/gkm952 PMC2238936

[B29] Altschul SF Gish W Miller W Myers EW Lipman DJ Basic local alignment search tool J Mol Biol 1990 215 3 403 410 2231712 10.1016/S0022-2836(05)80360-2

[B30] Rice P Longden I Bleasby A EMBOSS: the European Molecular Biology Open Software Suite Trends Genet 2000 16 6 276 277 10.1016/S0168-9525(00)02024-2 10827456

[B31] Gantier MP Baugh JA Donnelly SC Nuclear transcription of long hairpin RNA triggers innate immune responses J Interferon Cytokine Res 2007 27 9 789 797 10.1089/jir.2006.0152 17892400

[B32] Svoboda P Di Cara A Hairpin RNA: a secondary structure of primary importance Cell Mol Life Sci 2006 63 7-8 901 908 10.1007/s00018-005-5558-5 16568238 PMC11136179

[B33] Morse DP Bass BL Long RNA hairpins that contain inosine are present in Caenorhabditis elegans poly(A)+ RNA Proc Natl Acad Sci USA 1999 96 11 6048 6053 10.1073/pnas.96.11.6048 10339539 PMC26833

[B34] Hofacker IL Fontana W Stadler PF Bonhoeffer S Tacker M Schuster P Fast Folding and Comparison of RNA Secondary Structures (The Vienna RNA Package) Monatshefte fur Chemie 1994 125 167 188 10.1007/BF00818163

[B35] Chang TH Horng JT Huang HD RNALogo: a new approach to display structural RNA alignment Nucleic acids research 2008 36 Web Server W91 96 10.1093/nar/gkn258 18495753 PMC2447718

[B36] Gilbert SD Stoddard CD Wise SJ Batey RT Thermodynamic and kinetic characterization of ligand binding to the purine riboswitch aptamer domain J Mol Biol 2006 359 3 754 768 10.1016/j.jmb.2006.04.003 16650860

