# Supplementary figures and images for: An enhanced computational platform for investigating the roles of regulatory RNA and for identifying functional RNA motifs
Source: BMC Bioinformatics. 2013 Jan 21;14(Suppl 2):S4. doi: 10.1186/1471-2105-14-S2-S4 (PMC3549854; doi:10.1186/1471-2105-14-S2-S4)

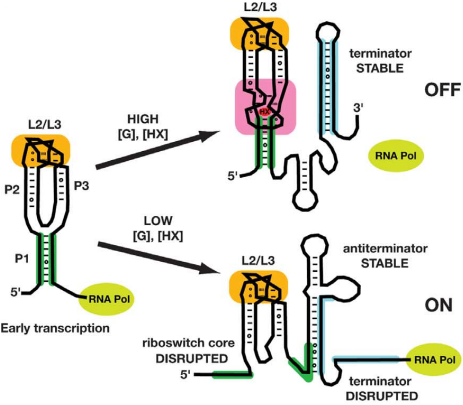

Supplement: Additional file 1 — A cartoon representation of the mechanism of genetic regulation by the guanine riboswitch [36]. [file 1471-2105-14-S2-S4-S1.png]
